# Efficacy of Prone Positioning in Non‐ARDS Patients With Hypoxemic Respiratory Failure: A Systematic Review and Meta‐Analysis

**DOI:** 10.1111/crj.70190

**Published:** 2026-05-14

**Authors:** Hany A. Zaki, Yavuz Yigit, Eman E. Shaban, Amira Shaban, Ahmed Shaban, Mohamed Gafar Abdelrahim, Mohamed Abosamak, Ali Elkandow, Karima Chaabna

**Affiliations:** ^1^ Department of Emergency Medicine Hamad Medical Corporation Doha Qatar; ^2^ College of Medicine Qatar University Doha Qatar; ^3^ Blizard Institute Queen Mary University London UK; ^4^ Department of Cardiology Al Jufairi Diagnosis and Treatment, MOH Doha Qatar; ^5^ Department of Internal Medicine Mansoura University Hospital Mansoura Egypt; ^6^ Department of Internal Medicine Mansoura General Hospital Mansoura Egypt; ^7^ Department of Anesthesia and Intensive Care Medicine, Faculty of Medicine Tanta University Tanta Egypt; ^8^ Institute for Population Health Weill Cornell Medicine‐Qatar Education City Qatar

**Keywords:** acute respiratory distress syndrome, hypoxemic respiratory failure, invasive ventilation, oxygenation, prone positioning

## Abstract

**Background:**

Prone positioning (PP) is established for the management of hypoxemic respiratory failure (HRF) due to acute respiratory distress syndrome (ARDS). However, its effectiveness in patients with HRF unrelated to ARDS remains unclear. This systematic review and meta‐analysis aimed to evaluate the clinical outcomes of PP in patients with non‐ARDS HRF.

**Methods:**

We systematically searched PubMed, Cochrane Library, Web of Science, and EMBASE (up to February 2025) for observational studies and randomized controlled trials (RCTs) evaluating PP in patients with HRF unrelated to ARDS. We included studies that enrolled either intubated or non‐intubated patients. On the other hand, we excluded studies that combined PP with other positions and those that did not compare pre‐and‐post prone or did not have control groups. Two reviewers working independently screened the included studies and extracted the relevant data. Meta‐analyses using a random‐effects model were performed using the Review Manager software.

**Results:**

Twenty‐one studies comprising 3040 patients with HRF unrelated to ARDS were included. Thirteen were observational studies, one was a non‐randomized clinical trial, and seven were RCTS. In non‐intubated patients, PP improved PaO2/FiO2 compared with baseline (mean difference [MD]: −51.36 mmHg; 95% CI: −70.39 to −32.32; *p* < 0.00001), decreased the need for intubation compared with control/standard care (risk ratio [RR]: 0.74; 95% CI: 0.60–0.91; *p* = 0.004), and demonstrated a trend to improve mortality (RR: 0.81; 95% CI: 0.61–1.01; *p* = 0.07). In intubated non‐ARDS HRF patients, the PaO_2_/FiO_2_ ratio was higher after PP, but not statistically significant (MD: −29.84; 95% CI: −77.51 to 17.83; *p* = 0.22).

**Conclusion:**

In non‐intubated patients with HRF unrelated to ARDS, PP improved oxygenation and decreased the need for invasive ventilation without increasing mortality risk. Therefore, PP is likely an effective therapy for non‐intubated patients with non‐ARDS HRF. Furthermore, evidence suggests that PP can improve oxygenation in intubated patients with HRF unrelated to ARDS.

## Graphical abstract

This meta‐analysis demonstrates that prone positioning (PP) significantly improves oxygenation and reduces the need for intubation in non‐intubated patients with hypoxemic respiratory failure (HRF) unrelated to ARDS. No significant mortality difference was observed between PP and standard care groups. PP remains a promising, non‐invasive strategy for optimizing respiratory support in non‐ARDS HRF, particularly in resource‐limited settings.

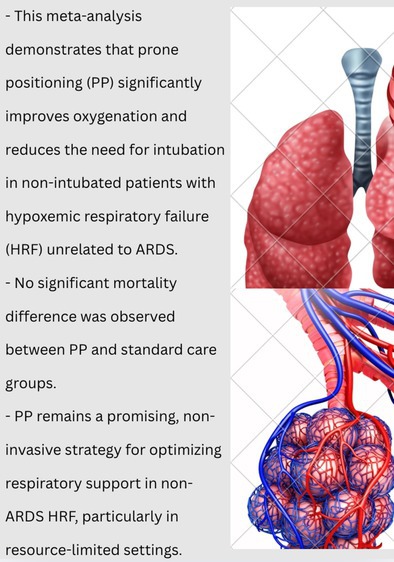

## 1 Introduction

Acute hypoxemic respiratory failure (AHRF) is one of the most common causes of admission to intensive care units (ICUs), with a reported fatality rate of approximately 30% [1, 2]. The majority of AHRF cases occur as a result of acute respiratory distress syndrome (ARDS), cardiogenic pulmonary edema, and pneumonia, but can also occur due to conditions such as coronavirus disease‐2019 (COVID‐19), chronic obstructive pulmonary disease, heart failure, and asthma [3, 4].

Traditionally, early AHRF is usually treated using non‐invasive ventilation (NIV). However, if NIV is ineffective, mechanical ventilation is often initiated. Prone positioning (PP) during mechanical ventilation has been used to treat severe hypoxemia in patients with ARDS for over 40 years [5]. This non‐pharmacological therapy has widely been adopted in ARDS patients following its positive impact on oxygenation and mortality [6, 7, 8]. The positive effect on oxygenation is often linked to mechanisms such as enhanced ventilation/perfusion matching, alleviation of compression in dependent lung areas due to the mediastinum's weight, and alterations in chest wall elastance [9, 10, 11]. Nonetheless, the efficacy of PP in non‐ARDS patients with AHRF is not well established. Therefore, by quantitatively examining the effects of PP on oxygenation, mortality, and intubation, this systematic review provides a comprehensive assessment of whether PP benefits patients with hypoxemia who do not meet ARDS criteria. In doing so, this study clarifies the role of PP beyond ARDS and informs clinical decision‐making in routine practice and future respiratory pandemics.

## 2 Methods

Our systematic review and meta‐analysis was developed based on the Cochrane Collaboration Handbook [11] and is reported as per the Preferred Reporting Items for Systematic Reviews and Meta‐analyses (PRISMA) guidelines [12]. The review protocol was preregistered in Open Science Framework https://doi.org/10.17605/OSF.IO/VBA89.

### 2.1 Literature Search and Information Sources

We conducted an extensive search on PubMed, Cochrane Library, Web of Science, and EMBASE for relevant studies published from databases' inception to February 2025. This search combined keywords related to two concepts: hypoxemic respiratory failure (HRF) and prone position. We also hand‐searched the reference lists of all included studies and previously published systematic reviews identified with the search. Furthermore, the search was limited to records authored in English. The complete database search strategy is shown in Appendix A.

### 2.2 Eligibility Criteria

We considered studies eligible for inclusion if they adhered to the following Population, Intervention, Comparison, Outcome (PICO) framework [13] criteria: **Participants (P):** Patients diagnosed with HRF secondary to conditions other than ARDS. **Intervention (I):** Prone position alongside any oxygen delivery system, including NIV, high‐flow nasal oxygen, simple oxygen therapy, and continuous positive airway pressure (CPAP). **Comparison (C):** Comparisons between pre‐ and post‐PP or between PP and standard care or no PP. **Outcomes (O):** Oxygenation, mortality, and intubation rates. Moreover, only studies enrolling at least 10 participants were included.

We excluded studies that did not adhere to the predefined PICO criteria and those reported as conference meeting abstracts, posters, editorials, case reports, narrative reviews, or letters to the editors, dissertations, and theses. In addition, we excluded studies that reported PP combined with lateral positioning.

### 2.3 Data Extraction and Data Items

Two investigators independently screened the title/abstracts and the full‐text records on Rayyan software. Conflicts were resolved by consensus.

Two investigators independently extracted the included studies on a standardized Excel spreadsheet. The information collected included study characteristics (e.g., the primary author, year of publication, study design, geographical location of the study, study setting [hospital department where the study was conducted]), patient characteristics (e.g., sample size, sex distribution, mean/median age, patient population, and etiology of AHRF), outcome (e.g., AHRF definition, mode of oxygen delivery, oxygenation, mortality, and/or intubation rates). The outcomes were categorized as primary or secondary, with the primary endpoint being oxygenation (PaO_2_/FiO_2_) and the secondary endpoints being mortality and intubation rates. We also extracted data related to the intervention (e.g., duration of PP) and comparator (e.g., standard/usual care).

### 2.4 Methodological Quality Assessment

Because we included both randomized controlled trials (RCTs) and observational studies, quality appraisal was undertaken using the Newcastle Ottawa Scale (NOS) [13] and the Cochrane Collaboration's risk of bias tool (ROB‐2) [14]. Using the ROB‐2 tool, RCTs were graded as having low, high, or unclear risk of bias across seven different domains, namely, random sequence generation, allocation concealment, blinding of participants and personnel, blinding outcome assessment, incomplete outcome data, selective reporting, and other bias. On the other hand, observational studies were graded as having poor, fair, or high methodological quality (NOS score between 0 and 3, 4 and 6, and 7 and 9, respectively). One reviewer performed this quality appraisal, and a second reviewer checked the quality appraisal to ensure consistency and completeness. Any conflicts in the quality assessment were resolved via constructive debates between the two reviewers or by soliciting the opinion of a third reviewer if an agreement was not met.

### 2.5 Data Synthesis

Meta‐analysis was performed using the Review Manager software (RevMan Version 5.4.1). For dichotomous outcomes (mortality and intubation), the overall effect size was estimated in terms of risk ratio (RR), which was computed with the Mantel–Haenszel method for binary data. For continuous outcome (oxygenation, assessed as changes in PaO_2_/FiO_2_ ratio between pre‐ and post‐PP) was reported in terms of mean difference (MD). For each of these analyses, a random‐effects model was employed to account for the expected variance. In addition, the pooled results were presented with their corresponding 95% confidence interval. Between‐study heterogeneity was assessed and quantified using the *I* ^2^ statistics, of which index values greater than 50% indicated significant heterogeneity [15, 16]. When significant heterogeneity was noted, we conducted subgroup meta‐analyses and random‐effects meta‐regressions to investigate the impact of the sources of heterogeneity related to study methodology, social determinants (e.g., Country and World Bank country‐income level), and population types (patients in different hospital departments, e.g., ICU, medical wards, and HDU). Publication bias was visually inspected using funnel plots and quantified using Egger's regression test. Both meta‐regression and Egger's test were conducted using the Comprehensive Meta‐Analysis (CMA Version 3.0) software. A significance level two‐tailed test was also used to determine whether the difference between the compared groups was significant, with a *p*‐value of less than 5% (*p* < 0.05) being the threshold of significance.

### 2.6 Dealing With Missing Data

For studies that did not report any outcome of interest (oxygenation, mortality, intubation, or adverse events), we reached out to the authors of the study and cross‐checked the data from closely related systematic reviews. If the missing data could not be obtained despite these attempts, the studies were excluded. When oxygenation data was presented in terms of the median and interquartile range or range, the mean and standard deviations were estimated using the formula presented by Wan and colleagues [17]. In addition, when oxygenation was presented as SpO_2_/FiO_2_ (SF) ratio, then conversion to PaO_2_/FiO_2_ (PF) ratio was done using the validated standard formula (SF = 57 + 0.61 PF) [18].

### 2.7 Certainty of Evidence

The Grading of Recommendations, Assessment, Development, and Evaluation (GRADE) approach was used to evaluate the certainty of evidence for the primary and secondary outcomes [19]. With the help of this tool, we graded outcomes as having high, moderate, low, and very low certainty based on five different domains, namely, the risk of bias, consistency of effect, imprecision, indirectness, and publication bias. The results of ROB‐2 and NOS were used to inform the grade for risk of bias, and the funnel plots and Egger's test informed the rating for publication bias.

## 3 Results

### 3.1 Search Results

**FIGURE 1:**
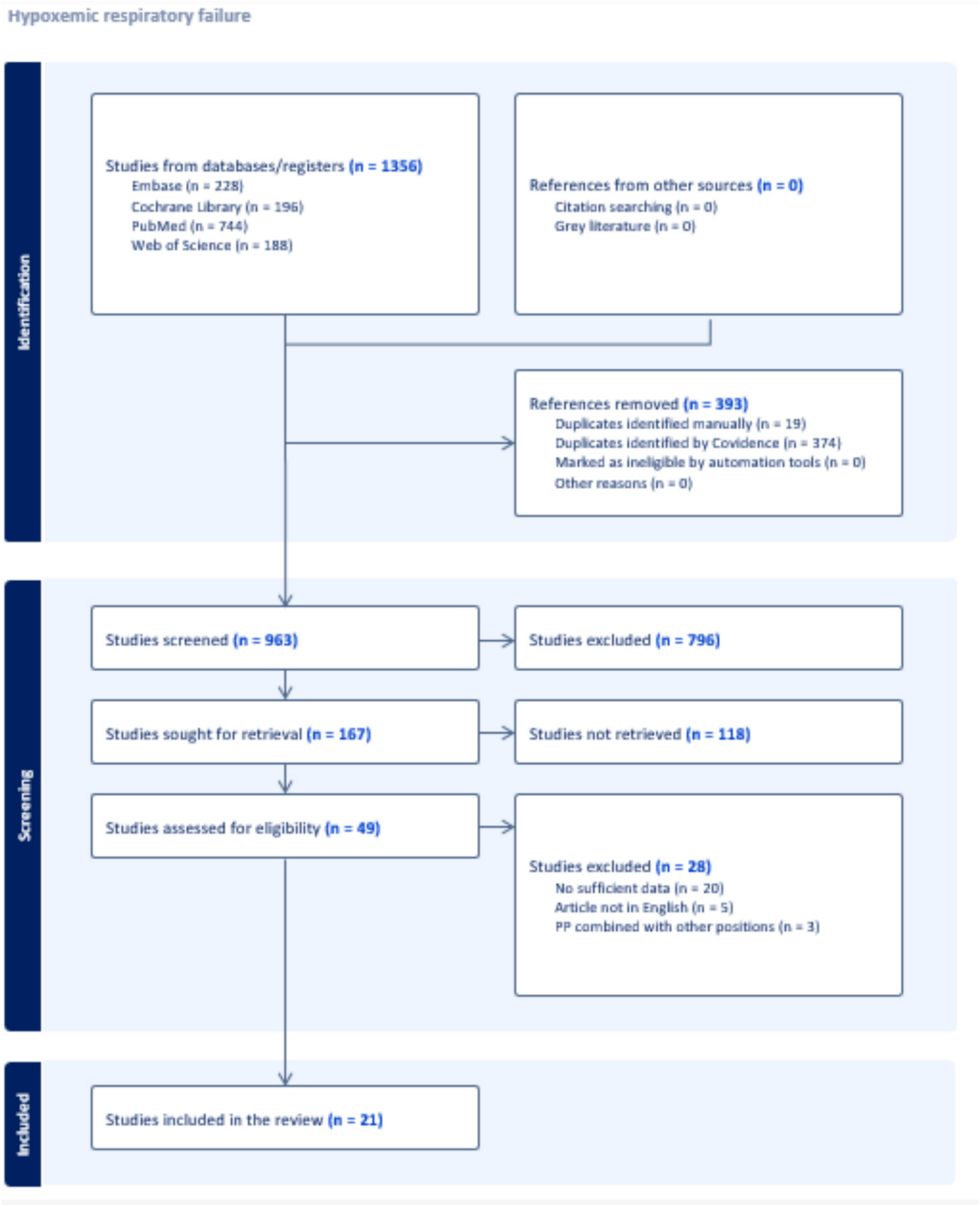
PRISMA flow diagram for study selection.

The comprehensive search strategy identified 1356 potential studies from databases and registers (Figure 1). A total of 393 duplicate records were excluded. An additional 796 records were excluded at the title/abstract screening stage and 49 full‐texts were screened. Finally, we included 21 primary studies.

### 3.2 Characteristics of the Included Studies

A total of 21 studies, comprising 3040 patients with HRF, were included in the quantitative analysis. Of these, seven were RCTs, one was a non‐randomized clinical trial, and 13 were observational studies. COVID‐19 pneumonia was the predominant cause of HRF, accounting for 2879 patients. Other reported etiologies included pneumonia, pancreatitis, fasciitis, chest trauma, and sepsis. Nineteen studies investigated the use of PP in non‐intubated patients, whereas two focused on intubated patients (Table 1).

**TABLE 1:** Summary of study characteristics.

Author ID	Study design	Study location	Setting	Patient characteristics					Respiratory support	Definition of AHRF	Duration of PP	Reported outcomes
				Sample (*n*)	M/F	Age (years)	Etiology of AHRF	Population				
Wang et al. [20]	Prospective observational study	China	ICU	10	7/3	68.5	Pneumonia (9) Pancreatitis (1)	Spontaneously breathing non‐intubated patients	HNFC	PaO_2_/FiO_2_ < 200 mmHg or SpO_2_/FiO_2_ < 235	30 min	SpO_2_/FiO_2_
Rana et al. [21]	Retrospective observational study	Pakistan	ICU or HDU	170	87/83	NR	COVID‐19 pneumonia	Non‐intubated patients	HNFC	6 L/min of oxygen to maintain SpO_2_ > 90%	8–10 h per day	SpO_2_/FiO_2_
Brunelle et al. [22]	Prospective crossover observational study	France	ICU	20	16/4	60	COVID‐19 pneumonia	Spontaneously breathing non‐intubated patients	NR	PaO_2_/FiO_2_: 100–300 mmHg	2 h with a washout period of 30 min	PaO_2_/FiO_2_
Bahloul et al. [23]	Prospective observational study	Tunisia	ICU	39	NR	61	COVID‐19 pneumonia	Spontaneously breathing non‐intubated patients	Face mask or HNFC	SpO_2_ < 92%	2–4 h (as tolerated)	Mortality and intubation
Lupieri et al. [24]	Retrospective observational study	Switzerland	ICU	31	23/8	60	COVID‐19 pneumonia	Intubated and non‐intubated patients	NR	PaO_2_/FiO_2_ < 200 mmHg	At least 45 min	PaO_2_/FiO_2_
Gad [25]	Prospective RCT	Egypt	Critical care isolation	30	17/13	NR	COVID‐19 pneumonia	Non‐intubated patients	Face mask or NIV	PaO_2_/FiO_2_ < 200 mmHg	1–2 h session with 3 h apart during waking hours	Mortality and intubation
Lehingue et al. [26]	Prospective, crossover RCT	France	ICU	17	15/2	61	COVID‐19 pneumonia	Spontaneously breathing non‐intubated patients	HNFC	PaO_2_/FiO_2_ ≤ 300 mmHg	2 h with a washout period of 2 h	PaO_2_/FiO_2_, intubation, and mortality
Syrma et al. [27]	Prospective observational study	India	NR	45	38/7	53.1	COVID‐19 pneumonia	Non‐intubated patients	HNFC, conventional oxygen therapy, or NIV	PaO_2_/FiO_2_ < 100 mmHg or room air SpO_2_ < 94%	2 h per section with a target of 8 h per day	Mortality and intubation
Fazzini et al. [28]	Prospective observational study	United Kingdom	Medical ward	46	NR	56	COVID‐19 pneumonia	Spontaneously breathing non‐intubated patients	HNFC, face mask, or CPAP	NR	≤ 1 h or > 1 h	PaO_2_/FiO_2_
Pierucci et al. [29]	Prospective observational study	Italy	ICU	32	23/9	NR	COVID‐19 pneumonia	Spontaneously breathing non‐intubated patients	HNFC, CPAP, or NIV	PaO_2_/FiO_2_ > 150 mmHg	72 h	PaO_2_/FiO_2_, intubation, and mortality
Koike et al. [30]	Retrospective observational study	Japan	ICU	58	43/15	67	COVID‐19 pneumonia	Spontaneously breathing non‐intubated patients	HNFC or NPPV	FiO_2_ ≥ 0.4	Until patient discharge	Mortality and intubation
Alhazzani et al. [31]	Pragmatic RCT	Canada, Kuwait, Saudi Arabia, and United States	ICU or acute care unit	400	283/117	NR	COVID‐19 pneumonia	Non‐intubated patients	NPPV or low/high‐flow oxygen devices	NR	Target duration of 8–10 h/day with 2–3 breaks of 1–2 h each	Mortality and intubation
Ehrmann et al. [32]	Prospective RCT	Canada, France, Ireland, Mexico, United States, and Spain	ICU, ED, or general ward	1121	746/375	NR	COVID‐19 pneumonia	Non‐intubated patients	HFNC	SpO_2_/FiO_2_ < 315 or PaO_2_/FiO_2_ ≤ 300 mmHg	As long and as frequently as possible every day	Intubation and mortality
Scaravilli et al. [33]	Retrospective observational study	Italy	ICU	15	10/5	58.3	Pneumonia (13) Fascitis (1) Sepsis (1)	Spontaneously breathing non‐intubated patients	Oxygen supply mask, HFNC, helmet CPAP, or NIV	PaO_2_/FiO_2_ ≤ 300 mmHg	6–8 h	PaO_2_/FiO_2_
Aisa et al. [34]	Prospective observational study	Ireland	ICU	50	23/27	56.2	COVID‐19 pneumonia	Spontaneously breathing non‐intubated patients	Conventional oxygen therapy, NIV, or HFNC	SpO_2_ < 90% or PaO_2_ < 10 kPa	Average of 8.5 h	PaO_2_/FiO_2_
Rosén et al. [35]	Prospective RCT	Sweden	Ward or ICU	75	55/20	NR	COVID‐19 pneumonia	Spontaneously breathing non‐intubated patients	HFNC or NIV	PaO_2_/FiO_2_ ≤ 20 kPa	Target duration of at least 16 h/day	Intubation and mortality
Musso et al. [36]	Open‐label, non‐randomized clinical trial	Italy	ICU	243	178/65	NR	COVID‐19 pneumonia	Non‐intubated patients	CPAP or PSV	PaO_2_/FiO_2_ ≤ 200 mmHg	1 session per day lasting ≥ 8 h overnight	Intubation and mortality
Estrada et al. [37]	RCT	Mexico	High acuity unit	430	258/172	NR	COVID‐19 pneumonia	Non‐intubated patients	HFNC	SpO_2_ < 90%	At least 1 h/day	Intubation and mortality
Venet et al. [38]	Prospective observational study	France	ICU	136	NR	NR	Pneumonia (103) Chest trauma (22) Pneumonia and chest trauma (11)	Mechanically intubated patients	NR	PaO_2_/FiO_2_ ≤ 300 mmHg	1 h	PaO_2_/FiO_2_
Bianchi et al. [39]	Prospective observational study	Italy	ICU	12	10/2	60	COVID‐19 pneumonia	Spontaneously breathing non‐intubated patients	CPAP, HFNC, or NIV	PaO_2_/FiO_2_ ≤ 200 mmHg	2 h	PaO_2_/FiO_2_
Jayakumar et al. [3]	RCT	India	ICU	60	50/10	NR	COVID‐19 pneumonia	Non‐intubated patients	Nasal prongs, face mask, non‐rebreather mask, HFNC, or NIV	PaO_2_/FiO_2_: 100–300 mmHg	At least 6 h/day	Intubation and mortality

### 3.3 Prone Position in Non‐Intubated Patients With Non‐ARDS HRF

**FIGURE 2:**
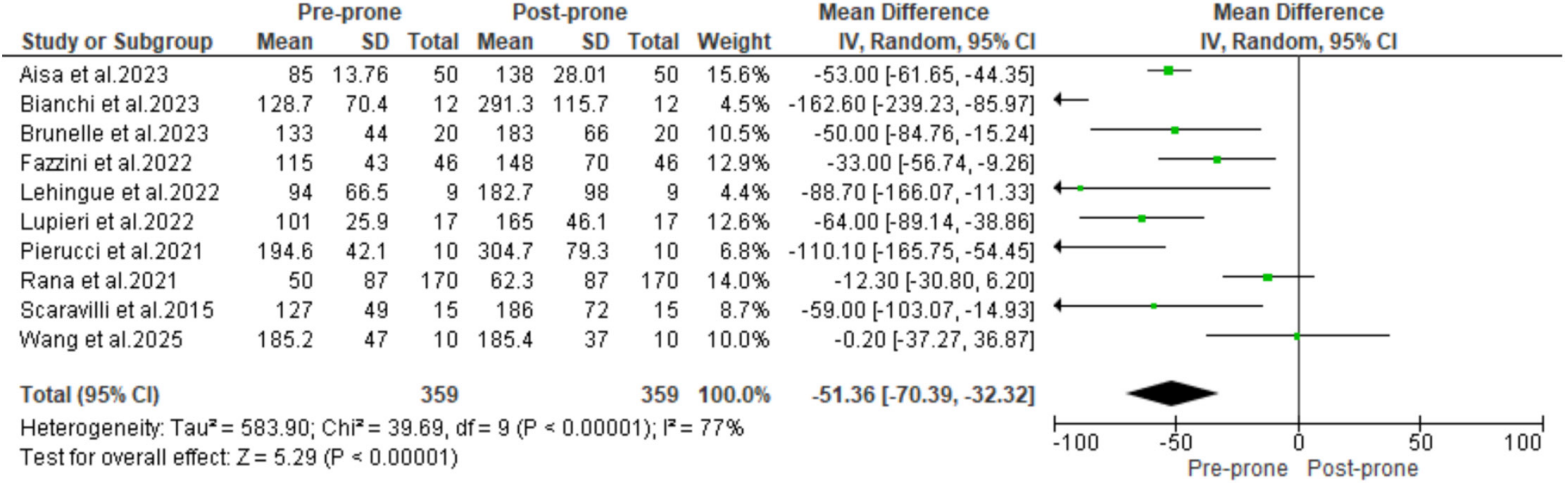
Change in PaO_2_/FiO_2_ ratio after prone positioning in non‐intubated patients.

**FIGURE 3:**
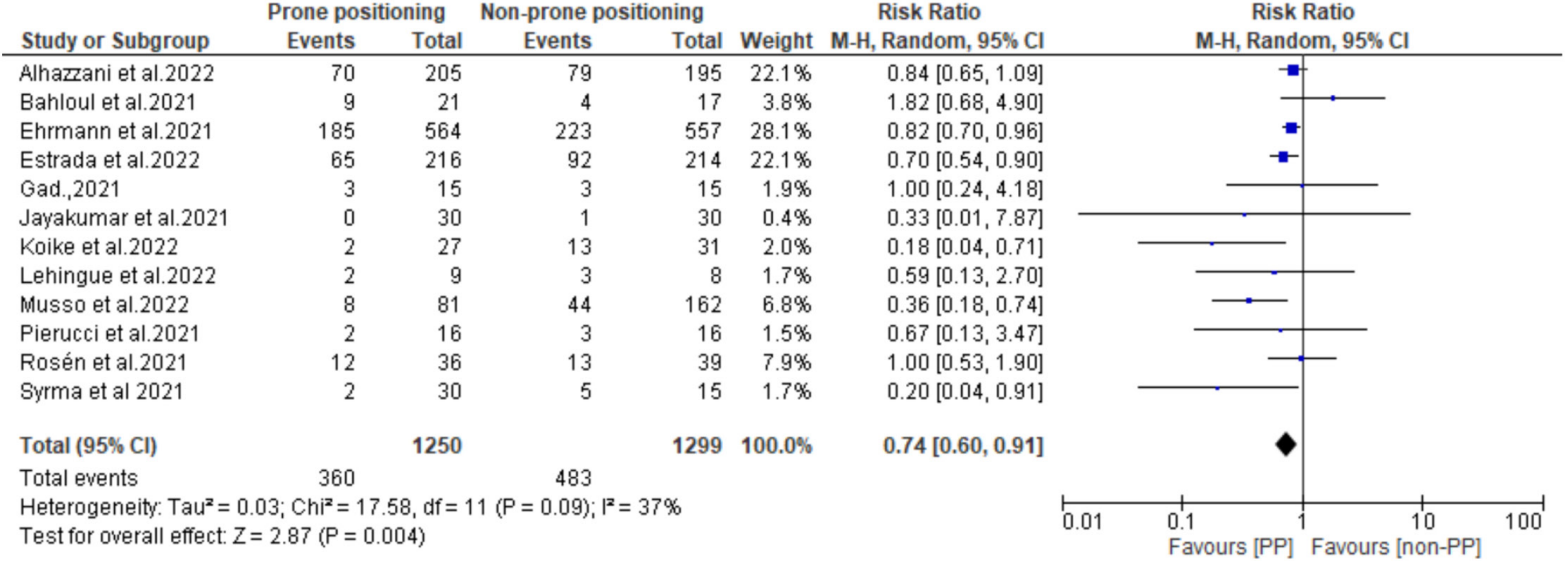
Intubation rates between prone and non‐prone groups.

**FIGURE 4:**
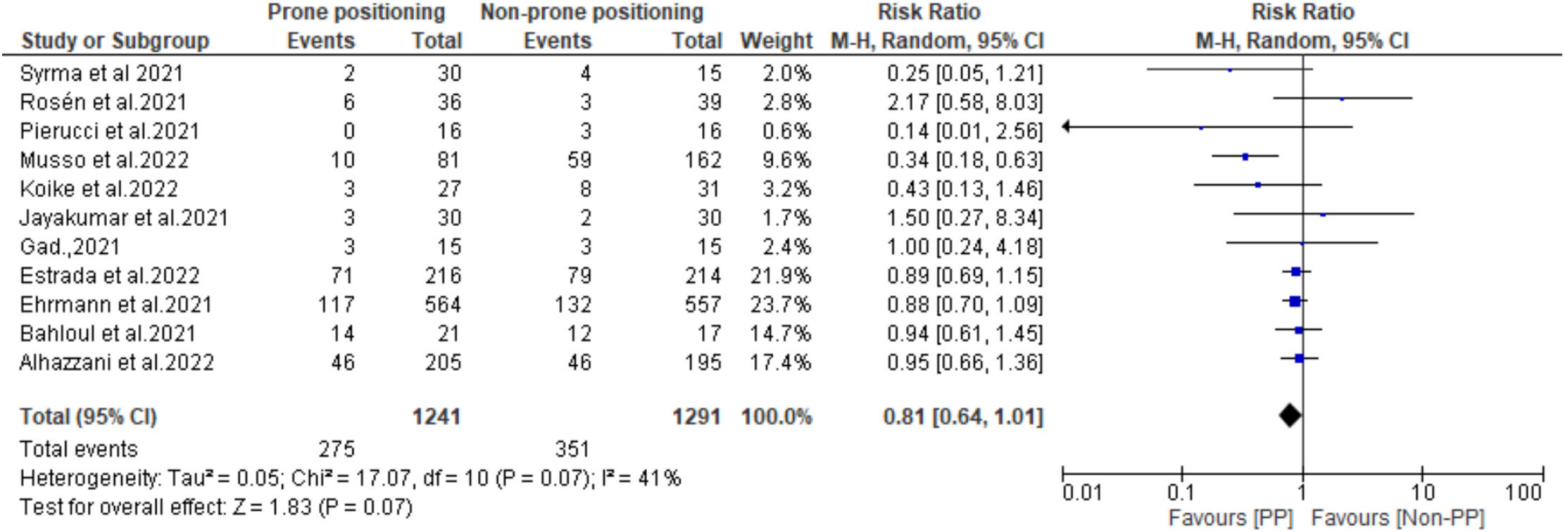
Mortality rates between prone and non‐prone groups.

Ten studies reported the impact of PP on oxygenation among non‐intubated patients with non‐ARDS HRF. Pooled data demonstrated that the PaO_2_/FiO_2_ ratio at the end of PP was significantly higher than before PP (MD: −51.36 mmHg; 95% CI: −70.39 to −32.32; *p* < 0.00001) (Figure 2), suggesting that PP improves oxygenation in this patient population. Additionally, the pooled analysis showed that the intubation rates were significantly lower in patients who underwent PP compared with those who did not (RR: 0.74; 95% CI: 0.60–0.91; *p* = 0.004) (Figure 3). The results also showed that mortality rates were lower in the PP group compared with the non‐PP group (275/1241 [22%] vs. 351/1291 [27%]). However, the difference between the two groups did not reach statistical significance (RR: 0.81; 95% CI: 0.61–1.01; *p* = 0.07) (Figure 4).

### 3.4 Prone Position in Intubated Patients With Non‐ARDS HRF

**FIGURE 5:**
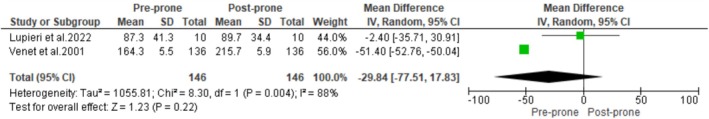
Change in PaO_2_/FiO_2_ ratio after prone positioning in intubated patients.

Only two included studies reported the use of PP in intubated patients. Data pooled from these studies showed that the PaO_2_/FiO_2_ ratio was greater after PP; however, the difference compared with before PP did not reach statistical significance (MD: −29.84; 95% CI: −77.51 to 17.83; *p* = 0.22) (Figure 5). Between‐study heterogeneity was significant and substantial (*I* ^2^ = 88%), largely driven by the small study by Lupieri et al. [24].

### 3.5 Quality Appraisal Outcomes

**FIGURE 6:**
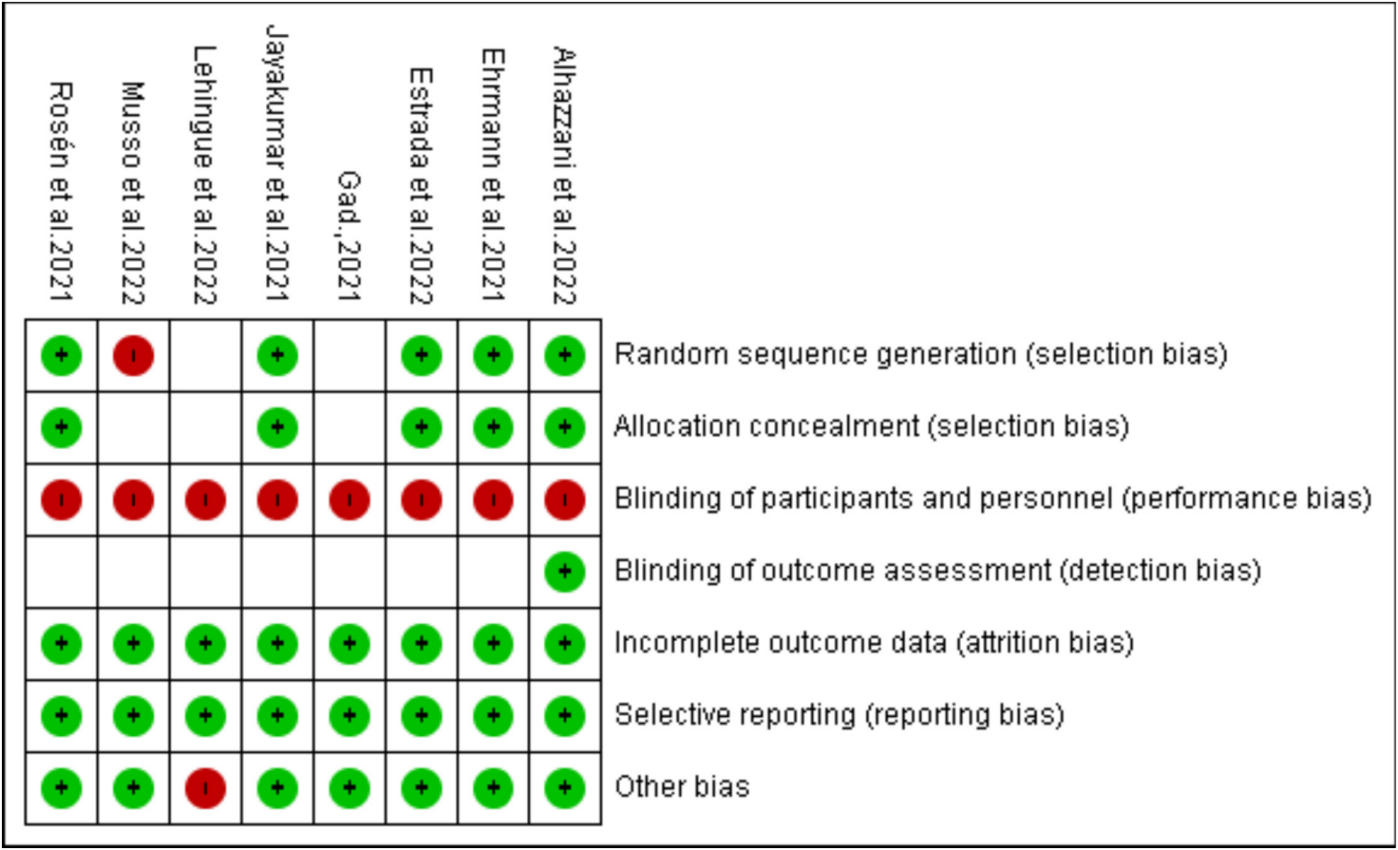
Risk of bias summary.

From the risk of bias summary (Figure 6), we can observe that all studies had a high risk of performance bias because blinding participants and personnel was impossible. One clinical trial was non‐randomized; therefore, it displayed a high risk of selection bias based on the lack of random sequence. In addition, one RCT demonstrated a high risk of other bias because it was designed as a crossover trial.

On the other hand, the overall NOS scores revealed that all studies had fair methodological quality. The most common reasons for the low methodological quality are as follows: First, all studies were carried out in single centers, which is not representative of the whole population. Second, given the nature of the intervention, blinding outcome assessors was impossible. Finally, none of the studies adjusted for the confounders (see Appendix B).

### 3.6 Subgroup Analyses and Meta‐Regression

Significant heterogeneity was only noted in oxygenation outcomes for both intubated and non‐intubated patients. However, due to a small number of studies evaluating oxygenation in intubated patients, we could not conduct subgroup and meta‐regression analyses. On the other hand, the subgroup and meta‐regression analyses showed that the World Bank country income level was a significant source of heterogeneity for oxygenation outcome in non‐intubated patients. Other factors, such as country, study design, and study setting, were not identified as significant sources of heterogeneity (Table 2).

**TABLE 2:** Subgroup and meta‐regression analyses investigating the sources of heterogeneity in oxygenation outcome among non‐intubated patients.

Category	Subgroup	No. of studies	Effect size (95% CI)	Meta‐regression *p*
Country	China	1	−0.20 (−37.27 to 36.87)	0.26
	France	2	−56.50 (88.21 to −24.79)	
	Ireland	1	−53.00 (−61.65 to −44.35)	
	Italy	3	−103.97 (−160.56 to −47.38)	
	Pakistan	1	−12.30 (−30.80 to 6.20)	
	Switzerland	1	−64.00 (−89.14 to −38.86)	
	United Kingdom	1	−33.00 (−56.74 to −9.26)	
World Bank country‐income level	High‐income	8	−61.60 (−78.67 to −44.54)	0.008
	Lower–middle income	1	−12.30 (−30.80 to 6.20)	
	Upper–middle income	1	−0.20 (−37.27 to 36.87)	
Study design	RCT	1	−88.70 (−166.07 to −11.33)	0.64
	Prospective observational studies	6	−54.75 (−80.65 to −28.85)	
	Retrospective observational studies	3	−43.10 (−81.76 to −4.43)	
Setting	ICU	8	−61.49 (−82.74 to −40.24)	0.14
	ICU or HDU	1	−12.30 (−30.80 to 6.20)	
	Medical ward	1	−33.00 (−56.74 to −9.26)	

### 3.7 Publication Bias

**FIGURE 7:**
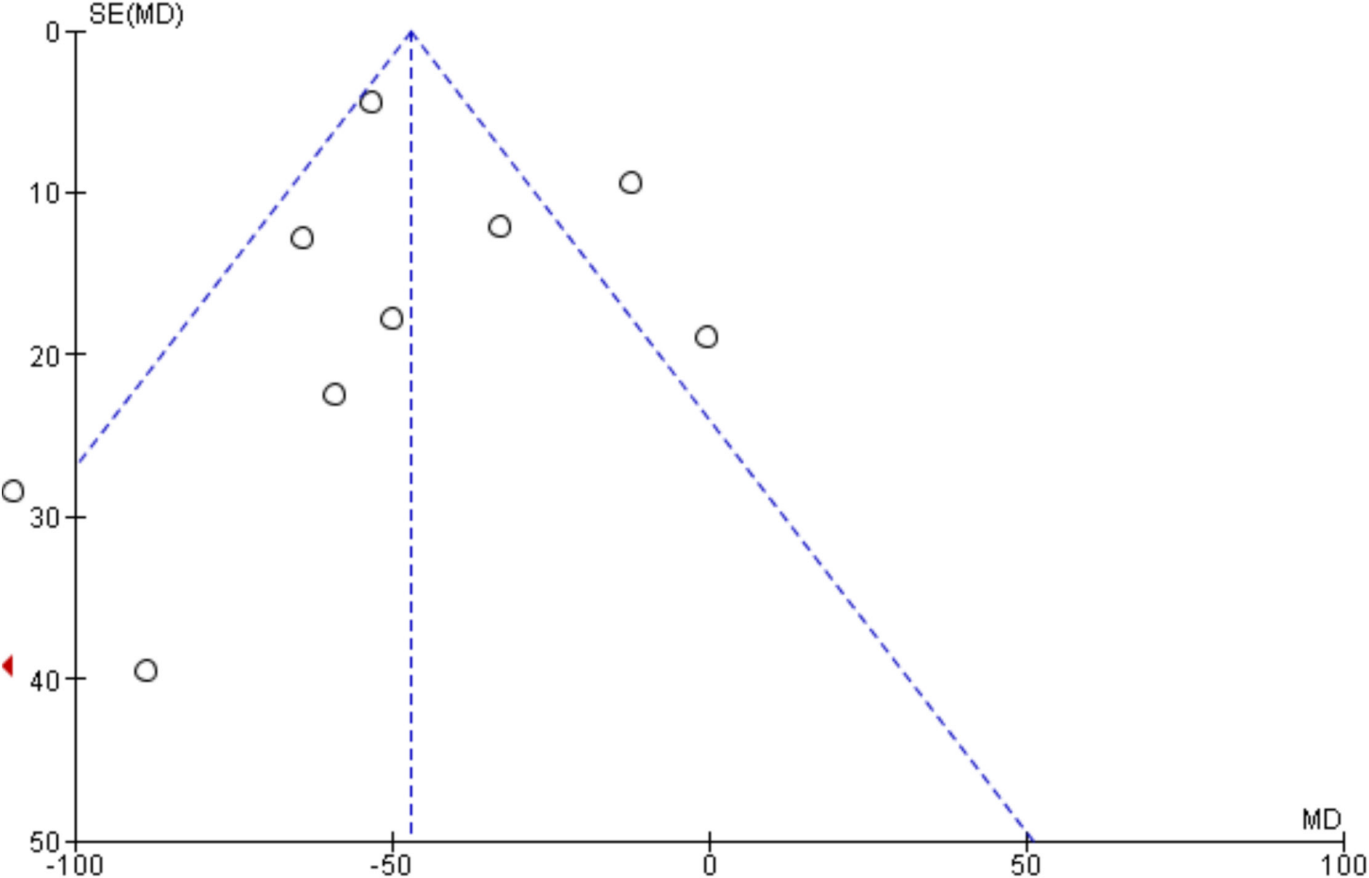
Funnel plot of change in PaO_2_/FiO_2_ ratio after prone positioning in non‐intubated patients.

**FIGURE 8:**
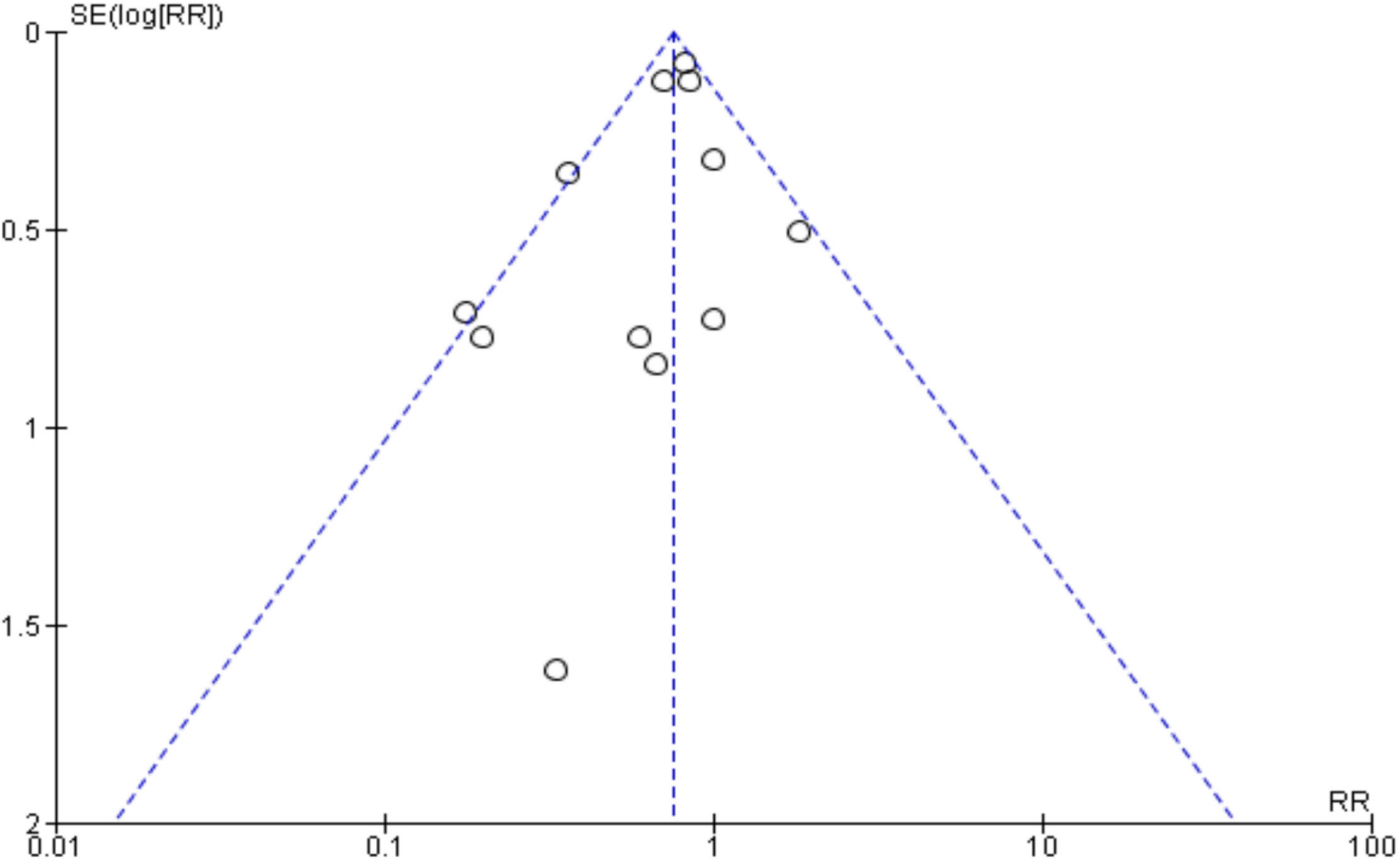
Funnel plot of Intubation rates between prone and non‐prone groups.

**FIGURE 9:**
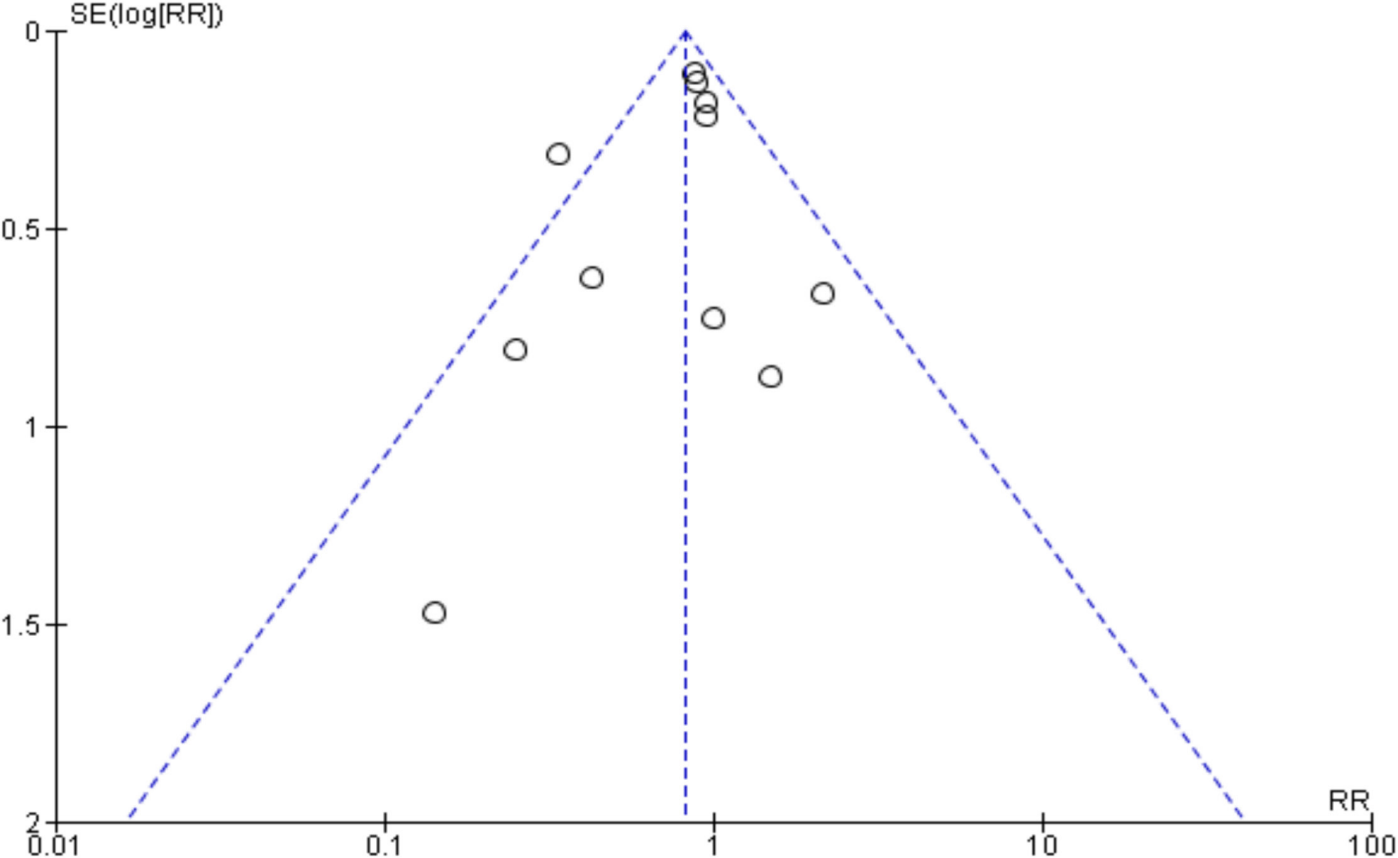
Funnel plot of mortality rates between prone and non‐prone groups.

**FIGURE 10:**
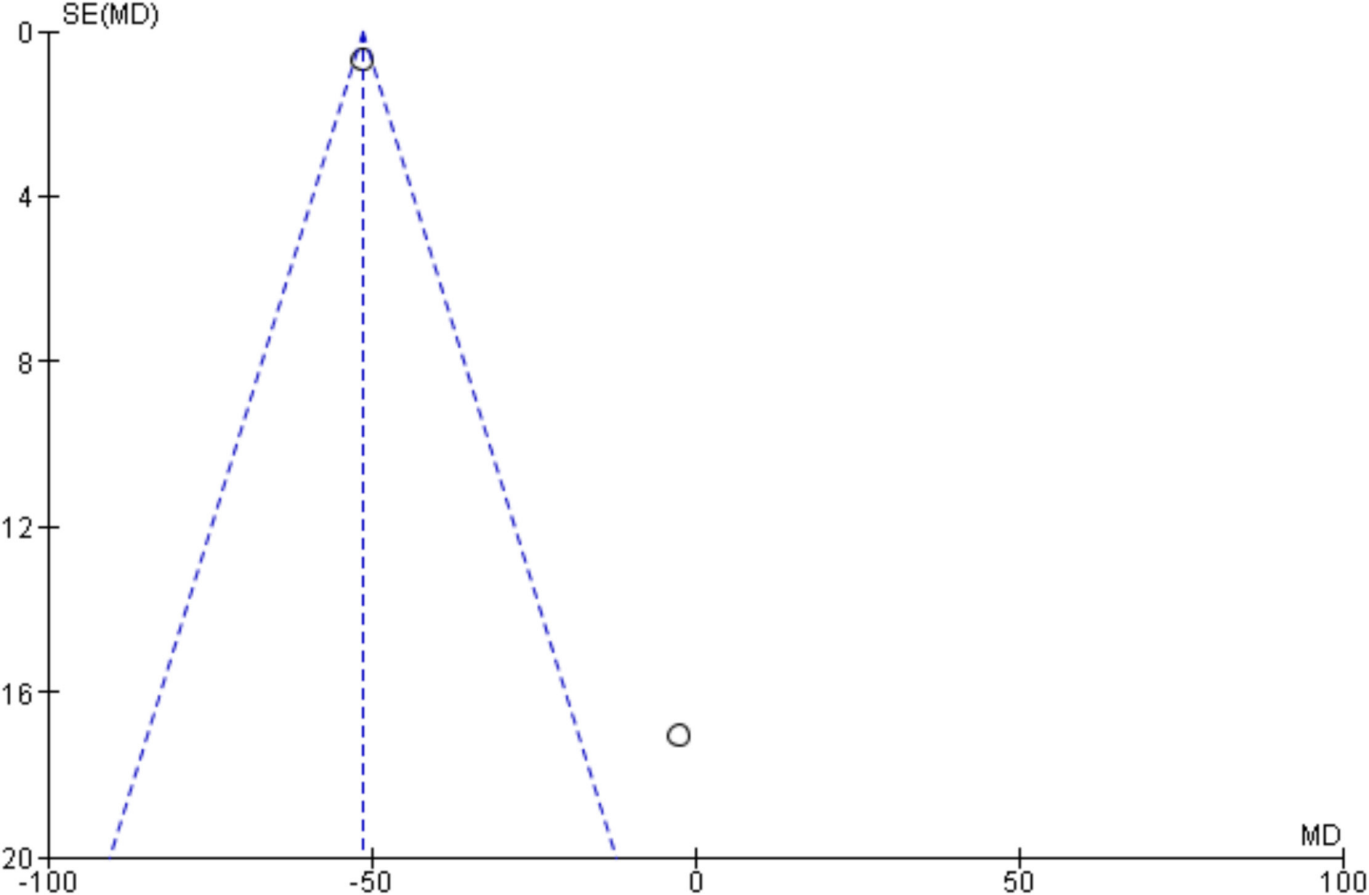
Funnel plot of change in PaO_2_/FiO_2_ ratio after prone positioning in intubated patients.

The funnel plots and Egger's regression test did not show any evidence of publication bias in oxygenation, intubation rates, and mortality outcomes among non‐intubated patients (Egger's regression test *p*‐values: 0.61, 0.22, and 0.33, respectively) (Figures 7, 8, 9). Similarly, we did not observe publication bias in oxygenation outcome among intubated patients (Figure 10).

### 3.8 Certainty of Evidence

The GRADE assessment demonstrated a low level of certainty for oxygenation, intubation rates, and mortality outcomes in non‐intubated patients. This low level of certainty was attributed to factors such as high heterogeneity, imprecision due to wide 95% CIs, and a large number of non‐randomized studies used in the analysis. The assessment also showed a very low level of certainty for the oxygenation outcome in intubated patients (see Appendix C).

## 4 Discussion

Although it is clear that PP improves oxygenation and lowers the mortality rate of mechanically ventilated patients with ARDS [8, 40], very few studies have assessed the efficacy and safety of PP in patients with non‐ARDS‐related HRF. Therefore, the current meta‐analysis reports the outcomes of PP in patients with HRF due to etiologies other than ARDS.

Our findings have shown that PP significantly improves oxygenation in non‐intubated patients with non‐ARDS‐related HRF. This finding aligns with a recently published meta‐analysis, which found that awake PP significantly improved oxygenation in non‐intubated patients with AHRF secondary to COVID‐19 [41]. This improved oxygenation after PP might be attributed to several mechanisms. For instance, PP can physiologically promote lung recruitment of the posterior dorsal regions by reversing atelectasis [42]. In addition, PP can result in more homogeneous ventilation by reducing lung strain caused by the variation in pleural pressure and pleural space distribution, thus improving ventilation/perfusion matching and lowering shunting [43, 44]. In contrast, one of the included studies enrolling patients with non‐COVID‐19 AHRF did not show a significant improvement in the SpO_2_/FiO_2_ ratio after PP [20], implying that PP does not improve oxygenation in patients with AHRF caused by conditions other than COVID‐19 and ARDS. However, this finding was limited by the study's small sample size and the fact that measurements were collected 30 min after the start of PP. Therefore, further research in large‐sample prospective studies with prolonged PP duration is needed to understand the exact effect of PP on oxygenation among patients with non‐COVID‐19 and non‐ARDS AHRF.

The current meta‐analysis has also shown that PP significantly reduces the need for intubation among non‐intubated patients with non‐ARDS HRF. This finding is consistent with three previously published systematic reviews, which support that awake PP is associated with a significant reduction in intubation rates among non‐intubated patients with COVID‐19‐related AHRF [41, 45, 46]. This improvement in intubation rates after PP can be attributed to the following reasons: First, most of the studies involved patients in the ICU, meaning that patients were more likely to receive intensive monitoring due to a higher healthcare workers‐to‐patient ratio in the ICU. Indeed, a previous meta‐analysis assessing the effect of PP in patients with COVID‐19‐related HRF found that PP was associated with reduced rates of intubations in ICU patients but not non‐ICU patients [41]. Second, as shown by our results, PP improves oxygenation, which might delay or prevent intubation. Finally, PP can delay or prevent progression to more severe respiratory failure, thus minimizing the need for intubation.

Although the pooled results revealed that PP improves oxygenation and reduces the risk of intubation in non‐intubated patients with non‐ARDS HRF, it was not associated with a significant decrease in mortality. This finding might be attributed to the fact that the improved oxygenation during proning is often temporary and does not persist after returning patients to the supine position. Indeed, Scaravilli and colleagues reported a significant improvement in PaO_2_/FiO_2_ ratio during PP, but the improvement significantly declined 6–8 h after returning patients to the supine position [33]. Nevertheless, evidence from some of the included studies has shown that PP is associated with lower mortality than standard care. For instance, a multivariable analysis by Musso and colleagues revealed that PP therapy was an independent predictor for mortality (HR: 0.2329; *p* < 0.001) and endotracheal intubation (HR: 0.1938; *p* < 0.001) [36].

Our analysis did not demonstrate a statistically significant improvement in oxygenation in intubated patients with non‐ARDS HRF undergoing PP. However, this finding was based on only two studies, both of which reported improvements in oxygenation. The lack of statistical significance was likely influenced by substantial heterogeneity, primarily driven by a small study. Notably, Venet et al. reported that PP during mechanical ventilation in non‐ARDS patients was associated with improved oxygenation [38]. Therefore, further research in large‐sample prospective studies is required to evaluate the potential benefits of PP in intubated patients with non‐ARDS HRF.

### 4.1 Strengths and Limitations

This meta‐analysis has several methodological strengths. Foremost, we conducted a comprehensive literature search across major databases to capture the full breadth of available evidence. The inclusion of both intubated and non‐intubated patients increased the applicability of our findings across a wide range of clinical settings. The use of random‐effects models strengthened our findings, given the expected clinical and methodological heterogeneity. Additionally, the inclusion of both RCTs and observational studies provides a broad and pragmatic evaluation of the current evidence base.

Nonetheless, this study has several limitations that should be acknowledged. First, substantial heterogeneity was noted in the pooled oxygenation outcomes, likely due to variation in sample size, study setting, duration of PP, study design, and/or geographical location. To address this, we used a random‐effects model. Second, the duration of prone sessions, patient selection criteria, and concurrent respiratory support likely varied across the studies, further driving heterogeneity. Third, given the nature of PP, blinding of participants and investigators was not feasible, potentially introducing bias. Fourth, most studies did not specify the exact duration of PP, limiting our ability to evaluate its impact on patient outcomes. Therefore, future studies should aim to systematically and accurately report PP start and end times. Fourth, the pooled dataset mixed observational studies and RCTs, which might have weakened causal inference. Fifth, the mortality trend observed in our study was statistically insignificant; thus, this finding should be interpreted cautiously and not taken as proof of mortality benefit. Sixth, selection bias may have been introduced as we included only studies published in English for inclusion. Seventh, the potential confounding in observational studies may have further contributed to selection bias and limited causal inference. Finally, most included studies focused on COVID‐19 pneumonia‐related HRF with only three addressing non‐COVID‐19‐related HRF. Therefore, additional studies on HRF unrelated to ARDS and COVID‐19 are needed to strengthen the evidence base.

### 4.2 Clinical Implications

From a clinical standpoint, our data indicate that PP is a viable, low‐risk strategy for non‐intubated patients with non‐ARDS HRF. In this group, PP may enhance oxygenation and decrease the need for endotracheal intubation, provided there is vigilant monitoring of patient tolerance and oxygenation response. Conversely, in intubated patients with HRF unrelated to ARDS, PP correlated with enhancements in oxygenation; yet, aggregated estimations failed to reveal a statistically significant impact on definitive clinical outcomes. Therefore, decisions about PP in intubated non‐ARDS patients must be tailored and informed by established ICU guidelines and the broader clinical context.

## 5 Conclusion

In this systematic review and meta‐analysis, PP improves oxygenation and reduces intubation rates, particularly in non‐intubated patients with non‐ARDS HRF. However, no statistically significant difference in mortality was observed between the PP and non‐PP groups, suggesting that PP does not increase or decrease mortality risk. Therefore, PP appears to be a feasible, safe, and effective non‐pharmacological intervention for managing non‐intubated patients with non‐ARDS HRF in medical ICUs, isolation rooms, or general wards. Moreover, its simplicity makes it especially valuable during pandemics (when the number of critically ill patients is relatively high) or when resources are limited, where reducing the need for invasive mechanical ventilation is critical. Although our results have shown that PP has no significant impact on oxygenation among intubated patients with non‐ARDS HRF, there is evidence suggesting that PP is still beneficial in terms of improving the oxygenation of these patients. Therefore, confirmatory trials involving intubated patients with non‐ARDS HRF are warranted.

## Author Contributions

H.A.Z. and E.E.S. conceived and designed the study. M.G.A., M.A. and A.E. performed literature search and data extraction. A.S., A.S. and K.C. contributed data analysis and interpretation. H.A.Z., Y.Y. and E.E.S. contributed to manuscript drafting and revision. K.C. provided methodological and statistical oversight. All authors reviewed and approved the final manuscript.

## Funding

The authors have nothing to report.

## Conflicts of Interest

The authors declare no conflicts of interest.

## Data Availability

The data that support the findings of this study are available from the corresponding author upon reasonable request.

## Appendix A Search Strategy

**PubMed**

**[All Fields]** (Prone position OR prone positioning OR Prone OR Proning) AND (acute respiratory failure OR hypoxemic respiratory failure OR hypoxemia OR acute hypoxemic respiratory failure OR hypoxia OR hypoxic respiratory Failure) AND (pneumonia OR cardiogenic edema OR COVID‐19 OR Coronavirus OR SARS‐CoV‐2), chronic obstructive pulmonary disease OR heart failure OR asthma OR non‐acute respiratory distress syndrome OR non‐ARDS)

**[Title/Abstract]** (Prone position OR prone positioning OR Prone OR Proning) AND (acute respiratory failure OR hypoxemic respiratory failure OR hypoxemia OR acute hypoxemic respiratory failure OR hypoxia OR hypoxic respiratory Failure) AND (pneumonia OR cardiogenic edema OR COVID‐19 OR Coronavirus ORSARS‐CoV‐2), chronic obstructive pulmonary disease OR heart failure OR asthma OR non‐acute respiratory distress syndrome OR non‐ARDS)

**EMBASE**

(Prone position OR prone positioning OR Prone OR Proning) AND (acute respiratory failure OR hypoxemic respiratory failure OR hypoxemia OR acute hypoxemic respiratory failure OR hypoxia OR hypoxic respiratory Failure) AND (pneumonia OR cardiogenic edema OR COVID‐19 OR Coronavirus OR SARS‐CoV‐2), chronic obstructive pulmonary disease OR heart failure OR asthma OR non‐acute respiratory distress syndrome OR non‐ARDS)

**Cochrane Library**

(Prone position OR prone positioning OR Prone OR Proning) AND (acute respiratory failure OR hypoxemic respiratory failure OR hypoxemia OR acute hypoxemic respiratory failure OR hypoxia OR hypoxic respiratory Failure) AND (pneumonia OR cardiogenic edema OR COVID‐19 OR Coronavirus OR SARS‐CoV‐2), chronic obstructive pulmonary disease OR heart failure OR asthma OR non‐acute respiratory distress syndrome OR non‐ARDS)

**Web of Science**

(Prone position OR prone positioning OR Prone OR Proning) AND (acute respiratory failure OR hypoxemic respiratory failure OR hypoxemia OR acute hypoxemic respiratory failure OR hypoxia OR hypoxic respiratory Failure) AND (pneumonia OR cardiogenic edema OR COVID‐19 OR Coronavirus OR SARS‐CoV‐2), chronic obstructive pulmonary disease OR heart failure OR asthma OR non‐acute respiratory distress syndrome OR non‐ARDS)

## Appendix B Quality Appraisal

**Table:** TABLE B1 Methodological quality appraisal using the Newcastle Ottawa Scale.

Study ID	Exposed cohort representativeness	Non‐exposed cohort selection	Exposure verification	Initial outcome absence	Cohort comparability (design/analysis adjusted for confounders)	Outcome evaluation	Sufficient follow‐up	Cohort follow‐up	Total score	Overall quality
Wang et al. [20]	0	0	1	1	0	0	1	1	4	Fair
Rana et al. [21]	0	0	1	1	0	0	1	1	4	Fair
Brunelle et al. [20]	0	0	1	1	0	0	1	1	4	Fair
Bahloul et al. [23]	0	1	1	1	0	0	1	1	5	Fair
Lupieri et al. [24]	0	1	1	1	0	0	1	1	5	Fair
Syrma et al. [27]	0	1	1	1	0	0	1	1	5	Fair
Fazzini et al. [28]	0	1	1	1	0	0	1	1	5	Fair
Pierucci et al. [27]	0	1	1	1	0	0	1	1	5	Fair
Koike et al. [30]	0	1	1	1	0	0	1	1	5	Fair
Scaravilli et al. [33]	0	0	1	1	0	0	1	1	4	Fair
Aisa et al. [34]	0	0	1	1	0	0	1	1	4	Fair
Venet et al. [38]	0	1	1	1	0	0	1	1	5	Fair
Bianchi et al. [39]	0	0	1	1	0	0	1	1	4	Fair

## Appendix C Certainty of Evidence

**Table:**

**Is prone position effective in non‐intubated patients with non‐ARDS HRF**					
**Patient or population:** Non‐ARDS hypoxemic respiratory failure **Setting:** **Intervention:** Non‐intubated patients **Comparison:** Placebo					
**Outcomes**	**No. of participants (studies) follow‐up**	**Certainty of the evidence (GRADE)**	**Relative effect (95% CI)**	**Anticipated absolute effects**	
				**Risk with placebo**	**Risk difference with non‐intubated patients**
Oxygenation	718 (10 non‐randomized studies)	⨁⨁◯◯ Low^a,b^	—	The mean oxygenation was **0**	MD **51.36 lower** (70.39 lower to 32.32 lower)
Intubation	2549 (12 non‐randomized studies)	⨁⨁◯◯ Low	**RR 0.74** (0.60–0.91)	372 per 1000	**97 fewer per 1000** (149 fewer to 33 fewer)
Mortality	2532 (11 non‐randomized studies)	⨁⨁◯◯ Low	**RR 0.81** (0.64–1.01)	272 per 1000	**52 fewer per 1000** (98 fewer to 3 more)
***The risk in the intervention group** (and its 95% confidence interval) is based on the assumed risk in the comparison group and the **relative effect** of the intervention (and its 95% CI). **CI:** confidence interval; **MD:** mean difference; **RR:** risk ratio					
**GRADE Working Group grades of evidence** **High certainty:** We are very confident that the true effect lies close to that of the estimate of the effect. **Moderate certainty:** We are moderately confident in the effect estimate: the true effect is likely to be close to the estimate of the effect, but there is a possibility that it is substantially different. **Low certainty:** Our confidence in the effect estimate is limited: the true effect may be substantially different from the estimate of the effect. **Very low certainty:** We have very little confidence in the effect estimate: the true effect is likely to be substantially different from the estimate of effect.					

*Explanations*

a. A high interstudy heterogeneity was observed.

b. The 95% CI was wide (more than 1).

**Table:**

**Is prone position effective for intubated patients with non‐ARDS hypoxemic respiratory failure**					
**Patient or population:** Non‐ARDS hypoxemic respiratory failure **Setting:** **Intervention:** Intubated patients **Comparison:** Placebo					
**Outcomes**	**No. of participants (studies) follow‐up**	**Certainty of the evidence (GRADE)**	**Relative effect (95% CI)**	**Anticipated absolute effects**	
				**Risk with placebo**	**Risk difference with Intubated patients**
Oxygenation	292 (2 non‐randomised studies)	⨁◯◯◯ Very low^a,b^	—	The mean oxygenation was **0**	MD **29.84 lower** (77.51 lower to 17.83 higher)
***The risk in the intervention group** (and its 95% confidence interval) is based on the assumed risk in the comparison group and the **relative effect** of the intervention (and its 95% CI). **CI:** confidence interval; **MD:** mean difference					
**GRADE Working Group grades of evidence** **High certainty:** We are very confident that the true effect lies close to that of the estimate of the effect. **Moderate certainty:** We are moderately confident in the effect estimate: the true effect is likely to be close to the estimate of the effect, but there is a possibility that it is substantially different. **Low certainty:** Our confidence in the effect estimate is limited: the true effect may be substantially different from the estimate of the effect. **Very low certainty:** We have very little confidence in the effect estimate: the true effect is likely to be substantially different from the estimate of effect.					

*Explanations*

a. A high interstudy heterogeneity was observed.

b. The 95% CI was wide.
